# Immunomolecular Characterization of MIC-1, a Novel Antigen in *Babesia bigemina*, Which Contains Conserved and Immunodominant B-Cell Epitopes that Induce Neutralizing Antibodies

**DOI:** 10.3390/vetsci5020032

**Published:** 2018-03-23

**Authors:** Diego Josimar Hernández-Silva, Uriel Mauricio Valdez-Espinoza, Miguel Angel Mercado-Uriostegui, Gabriela Aguilar-Tipacamú, Juan Alberto Ramos-Aragón, Ruben Hernández-Ortiz, Massaro Ueti, Juan Mosqueda

**Affiliations:** 1 Immunology and Vaccines Laboratory, Facultad de Ciencias Naturales, Universidad Autonoma de Queretaro, Av. de las Ciencias s/n Col Juriquilla, Queretaro 76230, Mexico; qfbjosimar@gmail.com (D.J.H.-S.); umvaldez@gmail.com (U.M.V.-E.); miguel.mercado.uriostegui@hotmail.com (M.A.M.-U.); 2 Facultad de Medicina Veterinaria y Zootecnia. Universidad Nacional Autonoma de Mexico. Av. Universidad 3000, Edificio A, Delegacion Coyoacan, Col. Ciudad Universitaria, Cd. Universitaria, Mexico City 04510, Mexico; 3 C.A. Salud Animal y Microbiologia Ambiental, Facultad de Ciencias Naturales, Universidad Autonoma de Queretaro, Av. de las Ciencias s/n Col Juriquilla, Queretaro 76230, Mexico; gabtipaca@yahoo.com.mx; 4 CENID-Parasitologia-INIFAP, Morelos 37915, Mexico; ramos.juan@inifap.gob.mx (J.A.R.-A.); hernandez.ruben@inifap.gob.mx (R.H.-O.); 5 Animal Disease Research Unit, USDA-ARS, 3003 ADBF, Washington State University, Pullman, WA 99164, USA; massaro@vetmed.wsu.edu

**Keywords:** bovine babesiosis, micronemal proteins, *Babesia bigemina*, sialic acid binding domain

## Abstract

*Babesia bigemina* is one of the most prevalent species causing bovine babesiosis around the world. Antigens involved in host cell invasion are vaccine targets for this disease but are largely unknown in this species. The invasion process of *Babesia* spp. into erythrocytes involves membrane proteins from the apical complex. A protein stored in the micronemes, called Micronemal Protein 1 (MIC-1), contains a sialic acid binding domain that participates in the invasion process of host cells and is a vaccine candidate in other apicomplexan parasites. It is not known if there is a homologous gene for *mic-1* in *B. bigemina*. Therefore, the aim of this study was to characterize the *mic-1* gene homologue in *Babesia bigemina*. A gene was found with a microneme adhesive repeat (MAR) domain in the predicted amino acid sequence. Transcription was determined by reverse transcription polymerase chain reaction (RT-PCR). Subsequently, antibodies against peptides containing conserved B-cell epitopes were used to confirm the expression of MIC-1 in intraerythrocytic merozoites. The presence of anti MIC-1 antibodies in cattle naturally infected with *B. bigemina* was determined and up to 97.4% of the cattle sera (113 out of 116) identified MIC-1 using enzyme-linked immunosorbent assay (ELISA) methods. Finally, antibodies against MIC-1 were able to block 70% merozoite invasion in-vitro.

## 1. Introduction

Bovine babesiosis is a tick-borne disease that is distributed in the tropical and subtropical regions of the world [1]. *Babesia bigemina* is one of the main etiological agents of the disease in the Americas causing an economic impact for the livestock industry [2,3,4]. 

During infection in the vertebrate host, the apicomplexan parasites, including *Babesia* species, use the proteins secreted by the apical complex organelles to invade the host red blood cells [5,6]. The proteins secreted by rhoptries, micronemes, and dense granules are the main molecules involved in invasion and escape from the host cells [6,7,8]. 

Microneme proteins (MICs) play a crucial role during red blood cell invasion. It has been suggested that they participate in the initial contact with the membrane of the host cell, followed by the reorientation of the apical complex and the release of proteins from rhoptries. The release of these proteins allows the parasite to penetrate the target cell [7,8,9]. MICs are key mediators for cell-cell interaction due to the adhesion domains they possess. Thus, they are considered adhesin proteins and are well conserved among the different species of apicomplexan parasites [5,10].

A sialic acid binding protein was identified in *Toxoplasma gondii* and it was named “protein secreted by the micronemes 1” (MIC-1). This protein contains 2 repeats of a sialic-acid binding microneme adhesive repeat (MAR) domain, arranged in tandem [11,12]. MIC-1 is essential for the anchorage of other MIC proteins to the membrane of the parasite during the process of host cell invasion and it has been considered as a vaccine candidate [12,13,14].

A *T. gondii* MIC-1 homologous sequence was found in the genome of *Babesia bovis*. The expression of the MIC-1 protein in *B. bovis* merozoites was demonstrated by indirect immunofluorescence. Importantly, antibodies against MIC-1 blocked in-vitro *B. bovis* invasion to red blood cells up to 95% [15]. 

There are no reports of micronemal proteins in *Babesia bigemina* described to date. Due to the importance of MIC-1 in the process of host cell invasion, the aim of this study was to identify and characterize the homologue of a MIC-1 protein in *B. bigemina*, to determine the existence of antibodies to this antigen in cattle naturally infected with *B. bigemina,* and to evaluate the capacity of specific antibodies to block red blood cell invasion. 

## 2. Materials and Methods 

### 2.1. Babesia bigemina Strains, Parasite Culture and Bovine Sera

*Babesia bigemina* strains from different geograpHical locations were used. Four strains were obtained from infected blood samples, two from Mexico (Tamaulipas and Chiapas) and two from Brazil (Rio Grande and Rondonia). Moreover, two strains were obtained from infected *Rhipicepalus microplus* ticks from Mexico (San Luis Potosi and Veracruz). A Puerto Rico strain that is maintained cultured in-vitro at Washington State University, was also included. All were used for DNA isolation and amplification.

The *B. bigemina* Puerto Rico strain was used for the neutralization assays. The merozoites were cultured in 96 well plates with HL-1 medium supplemented with 5% of bovine red blood cells, 40% of bovine serum, 0.1 M TAPSO, and pH was adjusted to 7.2. The culture was inoculated with an initial 1% parasitemia and the plates were incubated at 37 °C and 5% of CO_2_. Every 24 h 75% of total media volume was replaced with fresh media trying to not disturb the cells at the bottom of the well. When the parasitemia reached about 5%, the culture was split, fresh red blood cells and media were added and the parasitemia was adjusted again to 1%. This procedure ensured that fresh culture was available for the neutralization assay [3]. One hundred and sixteen field serum samples, confirmed as positive for *B. bigemina* by an indirect immunoflourescence antibody test (IFAT) [16], were used to evaluate the presence of specific anti-MIC-1 antibodies by indirect enzyme-linked immunosorbent assay (ELISA, see Table 1). 

### 2.2. Identification of mic-1 Gene in the Babesia bigemina Genome

In order to identify *mic-1* in the genome of *B. bigemina*, a Basic Local Alignment Search Tool (BLAST) analysis was performed using the reported sequence of *mic-1* gene from *B. bovis* (Genbank accession number: FJ613639) as a query to search in the database of the Sanger Institute (http://www.sanger.ac.uk).

### 2.3. Isolation of DNA and Amplification and Sequencing of B. bigemina mic-1

DNA from infected red blood cells was isolated according to the protocol described by Bartlett and Stirling [17]. DNA obtained from whole infected ticks was purified according to Mosqueda 2010 [16]. The DNA was quantified and kept at −20 °C until used for the polymerase chain reaction (PCR) amplification. The *mic-1* gene of each strain was amplified by PCR using two oligonucleotides, which amplified a 357 bp fragment from nucleotide 377 to 733 in the DNA sequence: BbigMIC-1 F 5′-CAC CGC TTC GAC GGA AAT GTG TC-3′ and BbigMIC-1 R 5′-ATG CCT TCA CCA CAG ATC CTA TCC -3′. The conditions of PCR were as follows: an initial denaturing step at 95 °C for 5 min, followed by 10 cycles at 94 °C for 30 s, 54 °C for 30 s and 72 °C for 30 s, followed by 20 cycles at 94 °C for 30 s, 57.5 °C for 30 s and 72 °C for 30 s, with a final extension step at 72 °C for 15 min. The amplicons were visualized by 2% agarose gel electropHoresis followed by EtBr staining. Each amplicon was purified using the Wizard^®^ SV Gel and PCR Clean-Up System (Promega, Madison, WI, USA), and immediately cloned into the pENTR^™^ Directional TOPO^®^ Cloning Kit (Thermo Fisher, Waltham, MA, USA) according to the manufacturer’s instructions. The inserts of the selected clones (3 of each strain) were sequenced by the dideoxy chain-termination method by the Biotechnology Institute of Universidad Nacional Autónoma de México (UNAM) in Cuernavaca, Mexico. The sequences were deposited in the GenBank under the accession numbers: Chiapas-Mexico strain MG775641, Veracruz-Mexico isolate MG775642, San Luis Potosi-Mexico isolate MG775643, Tamaulipas-Mexico isolate MG775644, Rio Grande-Brasil isolate MG775645, Rondonia-Brazil isolate MG775646. 

### 2.4. B. bigemina mic-1 Sequence Analysis

The *B. bigemina mic-1* sequences obtained were assembled and used to obtain each consensus sequence using the CLC Main Workbench software v.6.8 (Qiagen, Hilden, Germany). Each sequence was analyzed in silico: the number of introns and exons were predicted with the GENSCAN online tool (http://genes.mit.edu/GENSCAN.html). The predicted amino acid sequences were translated (http://web.expasy.org/translate/) and all the protein sequences were aligned using the Clustal Omega algorithm (http://www.ebi.ac.uk/Tools/msa/clustalo/) to identify regions within the MIC-1 protein sequences that were conserved in all the strains. In order to locate the extracellular domain, each sequence was examined to predict hydropHobic regions by Protscale (web.expasy.org/protscale/) [18], the signal peptide by SignalP (www.cbs.dtu.dk/services/SignalP/) [19], the transmembrane regions by TMHMM Sever v.2.0 (www.cbs.dtu.dk/services/TMHMM/) [20], and the protein domains were identified by Pfam (pfam.xfam.org/) [21] and SMART (http://smart.embl-heidelberg.de/). In order to find the peptides with high probability to be B cell epitopes, the conserved extracellular region of the protein was evaluated with four different algorithms: Antigenic (http://emboss.bioinformatics.nl/cgi-bin/emboss/antigenic), ABCpred (crdd.osdd.net/raghava/abcpred/) [22], BCEpred (crdd.osdd.net/raghava/bcepred/) [23], and IEBD (www.iedb.org/) [24]. The peptides with the highest scores in all four algorithms and that were conserved in all of the sequenced field isolates were selected for further experimental procedures. Finally, the selected peptides were analyzed by BLAST to confirm that they had 100% identity only with the corresponding sequence from *B. bigemina* and were not present in bovine nor in *B. bovis* proteins. Three peptides were selected and chemically synthesized in a multiple antigenic peptide system of eight branches (MAP8) by GL Biochem Company (Shanghai, China).

### 2.5. mic-1 Gene Transcription Analysis

In order to confirm gene transcription, a reverse transcription experiment was performed. For this, total RNA from *B. bigemina* infected bovine blood was isolated using Trizol Reagent following the manufacturer instructions (invitrogen, Carlsbad, CA, USA) as previously described [25]. The isolated total RNA was treated with DNase to avoid DNA contamination. The in-vitro cDNA synthesis was carried out using 100 ng/µL of RNA as a template and SuperScript II Reverse Transcriptase (Invitrogen, Carlsbad, CA, USA). Specific primers were designed that amplified a fragment of 223 bp, corresponding to a fragment from nucleotide 291 to 513 in the mRNA sequence. The primers used were F 5′-CGA GTA CTG TGC AGC GAC AAA C-3′ and R 5′-GTG GTG TAA CCT CTT TAT GAC GC-3′, the thermocycling protocol consisted of 5 min at 95 °C initially, 95 °C at 30 s followed by 35 cycles of 30 s at 65 °C, and 30 s at 72 °C, followed by 15 min at 72 °C. A sample of total RNA without reverse transcriptase was used as a control for DNA contamination. The presence of the amplicons was confirmed by electropHoresis in 3% agarose gels with ethidium bromide dye.

### 2.6. Anti-MIC-1 Antibody Production

To obtain anti *Babesia bigemina* MIC-1 antibodies, each synthetic peptide was inoculated subcutaneously into rabbits. For this, 100 µg of each peptide was resuspended in 500 µL PBS 1× and 500 µL Montanide ISA 71G (Seppic, Paris, France). Two two-month old New Zealand rabbits were inoculated with a single peptide (two rabbits for each peptide) six times every two weeks following the UNAM’s Bioethics Department procedures (SICUAE) with the approval number for immunization and sera analisis: MC/2014-38. Eleven days after the last immunization blood samples were taken from the central ear artery without anticoagulant. The sera collected, including the pre-immunization sera, were stored at −20 °C until used. Two rabbits were immunized only with adjuvant and PBS without peptide as a source of control sera. 

### 2.7. MIC-1 Protein Expression Analysis

#### 2.7.1. Western Blot

To evaluate the expression of MIC-1 in *B. bigemina* merozoites, first, a western blot analysis was carried out. Protein samples containing the lysates of infected erythrocytes of the Chiapas isolate of *B. bigemina* were suspended in loading buffer, in a 1:1 proportion, which were then boiled for 10 min previous to separation. The electropHoresis was performed at 110 V for 90 min in a running buffer (25 mM Tris Base, 200 mM Glycine, 3.5 mM SDS, pH 8.3) and the molecular weight marker from Precision Plus Protein™ Dual Color Standards was used (Bio Rad, Hercules, CA, USA). The separated proteins from the denaturing polyacrylamide gel were transferred to a nitrocellulose membrane (Bio Rad, Hercules, CA, USA) at 4 °C temperature at 100 V for 60 min, using transfer buffer (25 mM Tris base, Glycine 200 mM, Methanol 20%, pH 8.3). The transfer was verified with a Ponceau red stain (Ponceau red at 0.01%, acetic acid at 5%). The membrane was washed with TBS 1× (10 mM Tris Base, 150 mM NaCl) and then it was blocked using a blocking buffer (TBS 1×, 5% skim milk) overnight at 4 °C with shaking (420 rpm). The next day, the membrane was washed twice with TTBS 1× for 20 min at 400 rpm, and an additional wash with TBS 1× for 10 min. The membranes were incubated with the primary antibody (pre and post-immunization sera) at a 1:400 dilution for 60 min at room temperature, followed by washes of the membrane with 1× TBS. The membranes were incubated with the secondary antibody (anti-rabbit IgG conjugated with alkaline pHospHatase, Santa Cruz Biotechnology, Dallas, TX, USA) diluted at 1:1000 under the same conditions. The dilutions were prepared using 1× TTBS (TBS 1×, Tween20 0.05%). The immunodetection was achieved by using 5-bromium-4-chloride-3 indolyl pHospHate (BCIP) in combination with nitro blue tetrazolium (NBT) (Promega, Madison, WI, USA).

MIC-1 expression was also evaluated by an IFAT analysis using anti-MIC-1 antibodies. Briefly, smears with *B. bigemina*-infected bovine red blood cells, were fixed in acetone and incubated with a blocking buffer (donkey serum 5% diluted in PBS) at 37 °C for 10 min. The primary antibody (pre and post-immunization serum) was used at a 1:20 dilution, and the secondary antibody (donkey IgG anti-rabbit IgG conjugated with Alexa Fluor 488^®^ from Jackson ImmunoResearch, Baltimore, MD, USA) was diluted at 1:100, both of which were diluted in blocking solution. The incubations were done at 37 °C for 30 min in an incubator with wet paper towels. The smears were read with a fluorescence microscope, DM2500 model (Leica, Wetzlar, Germany) with 100× lens, using a light filter for 480 nm wave length.

#### 2.7.2. Evaluation of the Presence of Anti-*Babesia bigemina* MIC-1 Antibodies in Cattle from Endemic Areas

An indirect ELISA test was carried out to evaluate if cattle generate antibodies against MIC-1 during a natural *B. bigemina* infection. Bovine sera from different geographic locations and all positive to *B. bigemina* infection but negative to *B. bovis* by IFAT were used in this assay. To allow the detection of primary antibodies contained in the sera samples, MIC-1 peptides were bound to the wells of ELISA plates (flat bottom polystyrene high bind microplates, Corning, New York, NY, USA). Each well was filled with 100 µL of each MIC-1 peptide (10 µg/mL) suspended in carbonate buffer and incubated overnight at 4 °C. Three washes (PBS 1×, 0.05% Tween 20) were done to discard all the unbound peptides. The plate was blocked with 5% low-fat milk suspended in PBS pH 7.4 and then incubated at 37 °C for one hour. The wells were washed three times with a blocking solution, then 100 µL in a 1:40 proportion of each serum tested (primary antibody) was added and then incubated at 37 °C for an hour to allow antigen-antibody interaction. Unbound antibodies were eliminated by three washes. An HRP-conjugated anti-bovine IgG antibody (Jackson Immunoresearch, Baltimore, MD, USA) was used to detect the primary interaction and after an hour-incubation at 37 °C, the unbound secondary antibodies were eliminated by three washes. O-Phenylendiamine dihydrochloride (0.4 mg/mL) suspended in phosphate-citric acid buffer with hydrogen peroxide was used as substrate and the generation of colored product was measured with a 450 nm wave length ELISA reader after 20 min. Each sample was analyzed by triplicate and the threshold was calculated with the negative samples means plus three standard deviations. All samples below this value were considered as negative.

### 2.8. Neutralization Assay

To perform the neutralization assays, a *Babesia bigemina* in-vitro culture was grown until 6% parasitemia was reached. 1 × 10^6^ parasites contained in 16.5 µL were added to the fresh medium supplemented with bovine red blood cells and serum as described previously. The assay was performed in triplicate for each anti-MIC-1 antibody and the rabbit sera were added in 1:5 proportion in each replicate well. The proportion of rabbit serum in the culture was evaluated previously to avoid interference with the culture development; there was no statistical difference between control culture without rabbit serum and the culture tested with 1:5 serum proportion (data not shown). The culture was incubated at 37 °C in a 5% CO_2_ atmosphere for 48 h. After this period, the red blood cells were resuspended by pipetting up and down and an aliquot of the homogenized culture was taken to prepare smears. The parasitemia was calculated counting the infected and non-infected red blood cells in five representative fields [26].

### 2.9. Statistical Analysis

For neutralization assays, a comparative media analysis of non-paired samples was carried out to test significant differences within the cultures supplemented with pre-immunization and post-immunization serum with a *p* > 0.05. The data were analyzed using IBM Software SPSS version 22 [27].

## 3. Results

### 3.1. Identification of a mic-1 Homologous Gene in the Babesia bigemina Genome

**Figure 1:**
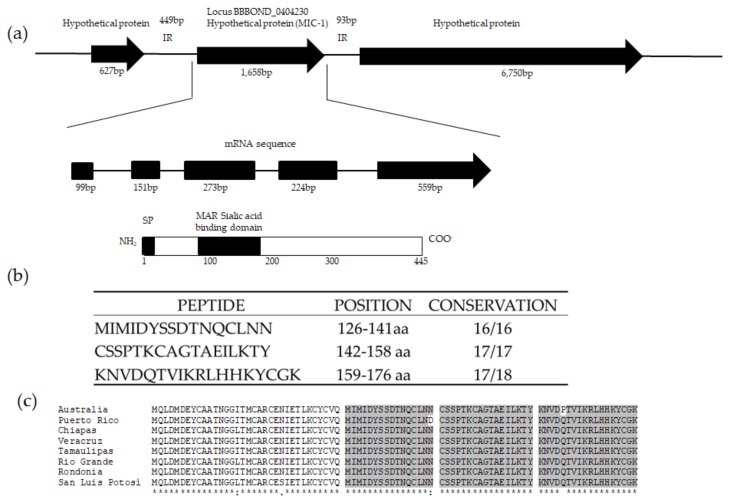
*Babesia bigemina micronemal protein 1* gene: (**a**) *mic-1* gene is localized in chromosome V as a hypothetical gene of 1658 bp (Accession number: XM_012914667). This gene contains 5 introns shown in the mRNA sequence as black bars. The product of *mic-1* is a protein of 445 amino acids with a predicted signal peptide that is cleaved in amino acid 23 and a microneme adhesive repeat (MAR) domain located from amino acid 85 to amino acid 172; (**b**) Selected MIC-1 peptides containing predicted B-cell epitopes; (**c**) Multiple alignment of the MAR domain of MIC-1 in 8 different isolates of *B. bigemina* indicating in grey the localization of the three selected peptides.

A BLAST search was performed using the *B. bovis mic-1* sequence as query. A contig in the genome of *B. bigemina* was identified containing a sequence that had a 32% similarity with *mic-1 of B. bovis*. According to the in silico analysis, the *mic-1* gene has a 1658 bp Open Reading Frame that codes putatively a 445 amino acid protein (Figure 1). The gene has five exons and four introns (Figure 1a). An mRNA sequence uploaded in the Genbank after our initial studies confirmed these findings. (Accession number: XM_012914667.1) The predicted peptide sequences translated from all the strains were aligned against the putative MIC-1 from the Australian strain. The analysis showed up to 97% of similarity in the amino acid sequence among all the strains analyzed.

MIC-1 is described as a secretory protein in other Apicomplexan parasites. The bioinformatics analysis performed here indicates a lack of transmembrane domains in the *B. bigemina* MIC-1 homologue. The predicted sequence also contains a signal peptide that is cleaved between amino acids 23 and 24 (Figure 1a). This information was used to select a fragment that remains intact in the mature protein. With the bioinformatics tools used here, we were able to identify three peptides that contain predicted B cell epitopes (Figure 1b). Through a multiple alignment of the amino acid sequences for the MAR domain, we demonstrated the conservation of these peptides among the different isolates of *B. bigemina*. Just for the case of the Puerto Rico isolate and the Australia isolate, a variation was observed in only one amino acid in the first and third peptide, respectively (Figure 1c). The peptides were named as MIC-1 A, MIC-1 B and MIC-1 C.

### 3.2. mic-1 Is Transcribed and Expressed in Blood Stages of Babesia Bigemina

**Figure 2:**
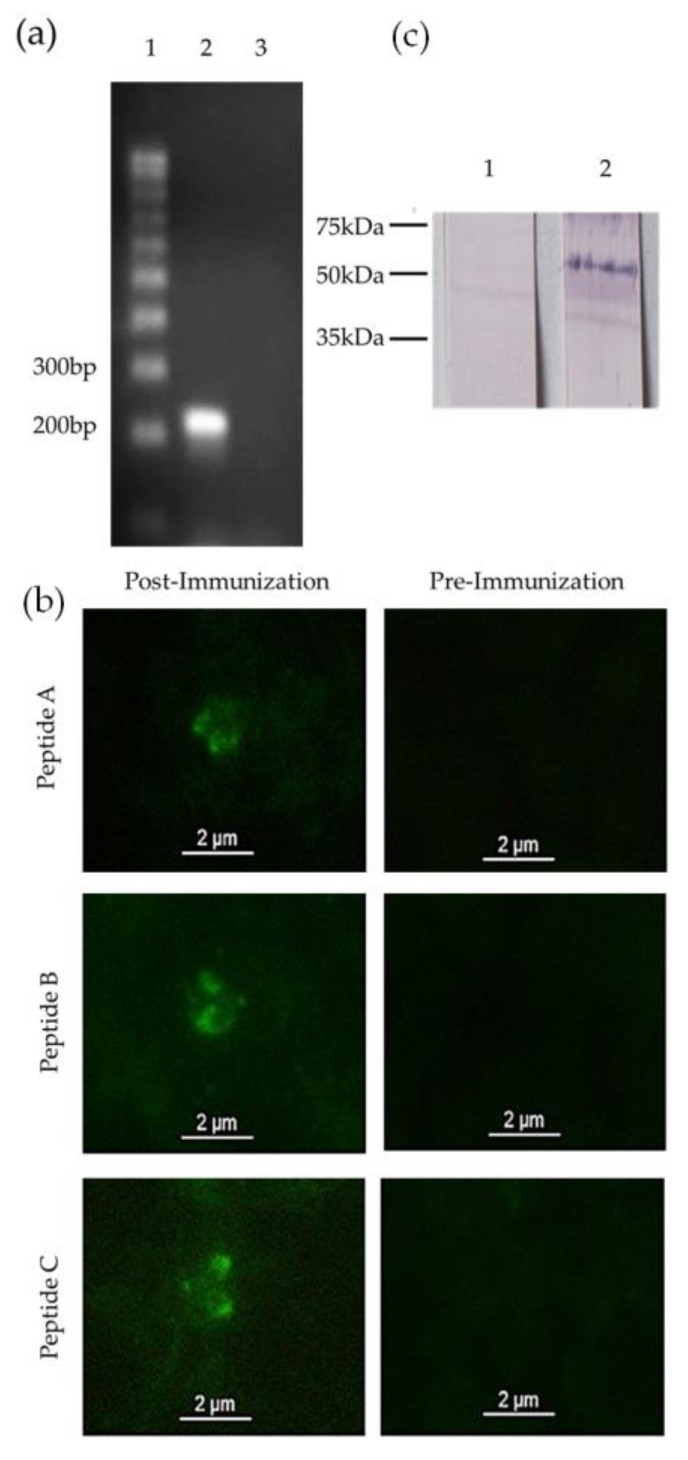
Micronemal Protein 1 is expressed in merozoites of *Babesia bigemina* (*B. bigemina*): (**a**) *mic-1* is a gene transcribed in blood stages of *B. bigemina*. Lane 1: Molecular weight marker. Lane 2: *B. bigemina* mRNA. Lane 3: *B. bigemina* mRNA without reverse transcriptase; (**b**) Intraerythrocytic merozoites of *B. bigemina* were incubated with sera from rabbits immunized with each MIC-1 peptide or with its corresponding pre-immunization serum. 100× objective; (**c**) Western blot analysis of MIC-1: Lane 1: infected red blood cells lysate incubated with pre-immune serum. Lane 2: Infected red blood cells lysate incubated with post-immune serum. Molecular markers on the left.

In order to determine if the *mic-1* gene was transcribed, a reverse transcription PCR was performed using specific primers for *B. bigemina mic-1*. The presence of transcripts was confirmed by obtaining an amplicon of the expected size in the mRNA sample (Figure 2a, lane 2). 

MIC-1 expression was analyzed by Indirect Immunofluorescence using *B. bigemina* merozoites. When the merozoites were incubated with post-immune sera from rabbits immunized with MIC-1, an intense signal in the anterior end of the merozoites was detected and the characteristic pear shape was clearly observed. The same stain pattern was observed when merozoites were incubated with antibodies against each of the three MIC-1 peptides (Figure 2b). There were no positive signals when merozoites were incubated with pre-immunization sera, confirming specific binding of the post-immune sera (Figure 2b). Finally, a Western Blot assay was used to corroborate the molecular size of MIC-1. Rabbit antiserum anti-MIC-1 recognized a protein band around 55 KDa in infected bovine red blood cells (Figure 2c). The rabbit pre-immunization serum did not recognize any proteins when it was incubated with infected red blood cell (Figure 2c).

### 3.3. Anti-MIC-1 Antibodies Are Present in Bovine Naturally Infected with Babesia Bigemina

To determine the presence of anti MIC-1 antibodies in cattle naturally infected with *B. bigemina*, 116 field serum samples from cattle in endemic areas and positive to *B. bigemina* infection were analyzed by indirect ELISA. The results indicate that 113 cattle out of 116 (97.4%) contained antibodies that recognized peptide MIC-1 A. On the other hand, 96 bovine sera (83%) recognized the MIC-1 B peptide (Table 1). However, all the cattle infected with *B. bigemina* had antibodies against at least one of the two MIC-1 peptides tested (Table 1). 

### 3.4. Antibodies against Babesia bigemina MIC-1 Block Parasite Invasion In-Vitro 

**Figure 3:**
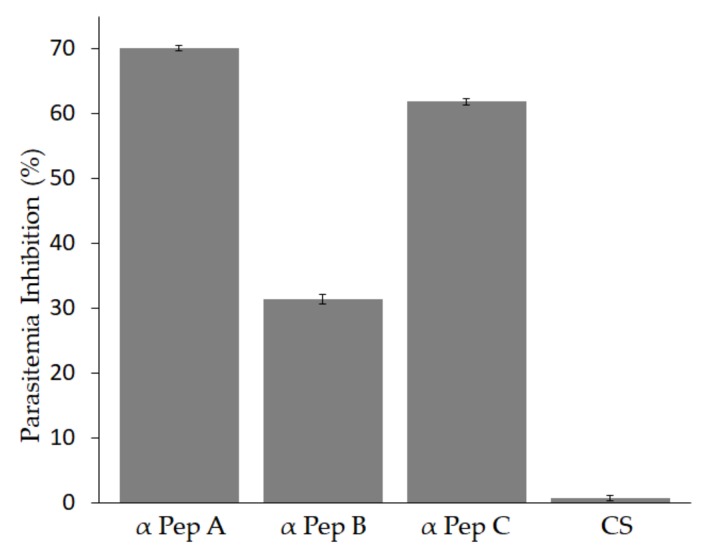
Effect of antibodies against *Babesia bigemina* MIC-1 to block parasite invasion in-vitro. Percentage of parasitemia inhibition in cultures supplemented with antibodies against each MIC-1 peptide. The percentage of infected erythrocytes was determined in *B. bigemina* cultures incubated with rabbit serum immunized with each anti-MIC-1 peptide. Serum from a rabbit immunized only with adjuvant (CS) was used as control. All data are expressed in mean percentage, the asterisk indicates the values that are significantly different from the control cultures incubated with pre-immunization serum (*p* < 0.05).

To determine whether antibodies against MIC-1 block parasite invasion, an in-vitro neutralization assay was performed. The results showed that when the *Babesia bigemina* culture was incubated with antibodies against each of the three MIC-1 peptides, a statistically significant difference was observed in the reduction of parasitemia in comparison to the culture supplemented with pre-immunization serum (Figure 3). The antibodies against MIC-1 A induced the highest reduction at 70%. The anti-MIC-1 C antibodies reduced the parasitemia by 61.85% and the anti-MIC-1 B showed the lowest neutralization activity at 31.40% (Figure 3). In-vitro cultures incubated with serum from a rabbit immunized only with adjuvant as control serum (CS) induced 0% reduction, indicating that the adjuvant did not have an effect on parasite growth in-vitro (Figure 3). 

## 4. Discussion

Due to the complex life cycle of *Babesia bigemina*, and the effect of the disease on the cattle industry, it is necessary to develop control measures against bovine babesiosis. Therefore, antigens involved in parasite invasion containing neutralizing, immunodominant B-cell epitopes, and conserved in geograpHically distinct strains, are required to be included in effective vaccines. In this work, we have demonstrated that *Babesia bigemina* contains the *mic-1* gene, an orthologue gene present in other species of *Babesia*, such as *Babesia bovis* [15]. Unlike *Bbo mic-1* described by Silva et al., the *B. bigemina mic-1* was found in chromosome V as a putative 1658 bp-long ORF (Accession number: XM_012914667) with five introns and a predicted protein product of 445 amino acids with a signal peptide cleaved at amino acid 23. Additionally, *B. bigemina* MIC-1 contains a predicted MAR Sialic Acid Binding Domain (located from amino acid 85 to amino acid 172). This domain is present in MIC-1 from other apicomplexan, like *Toxoplasma gondii* [28]. These distinctive features make *mic-1* a novel gene in *B. bigemina*. 

Here we demonstrate that *mic-1* is expressed both at the transcriptional and the protein level. When mRNA from intraerythrocytic parasites was retrotranscribed to cDNA and amplified with specific primers, we observed a single band of the expected size. Expression of the protein was confirmed by western blot, where a protein of approximately 55 kDa was detected in erythrocytes infected with *B. bigemina*, corresponding to the predicted size of MIC-1. The molecular weight of *B. bigemina* MIC-1 correlates with the MIC-1 protein of *T. gondii*, which is about 50 kDa [11]. Finally, we demonstrated the presence of MIC-1 in merozoites by indirect immunofluorescence, showing its localization in the anterior end of the parasite, corresponding to the localization of the micronemes in *Babesia* as it was demonstrated for *B. bovis* [15].

To determine if MIC-1 is involved in erythrocyte invasion and to evaluate the capacity of anti-MIC-1 antibodies to block invasion to host cells, an in-vitro culture assay was performed. We found a significant reduction in the percentage of parasitemia, with antibodies against each of the three peptides. Anti-MIC-1 antibodies reduced parasite invasion by 70% (MIC-1 A), 31.49% (MIC-1 B) and 61.85% (MIC-1 C). It is significant that in similar studies antibodies against *B. bovis* MIC-1 peptides reduced the parasitemia by 45.1% [15]. The neutralizing capacity of all the anti-MIC-1 antibodies evaluated in this study was statistically significant compared with the pre-immune serum or the serum from a rabbit immunized with adjuvant only. However, the difference in percentage of inhibition for each antiserum was large, and this could be related to the position of the peptide sequence in the protein, or even to the titer of the antiserum in each rabbit immunized, which was not evaluated in this work. Importantly, all three peptides were conserved in all the strains and induced reduction of parasitemia in-vitro, which could have implications for vaccine development. 

Finally, MIC-1 peptides were selected using regions of the protein that are conserved in different strains, therefore, it was important to investigate whether these conserved peptides containing B-cell epitopes generated antibodies in cattle naturally infected with *Babesia bigemina*. We found by an indirect ELISA that cattle naturally infected with *B. bigemina* produce antibodies that recognize MIC-1 A and MIC-1 B. We chose those two peptides for this evaluation, as they were the ones that produced the highest and the lowest inhibition of parasitemia in-vitro, as mentioned earlier. These findings are important as this indicates that these peptides contain immunodominant B-cell epitopes, and induce antibodies during a natural infection. It is also important to mention that 97.4% of the analyzed cattle contained antibodies to peptide A, while 83% of the cattle contained antibodies to peptide B. The difference between those results could be attributed to immunodominance. If peptide A is more immunodominant than peptide B, then it could explain why more bovines generated antibodies against peptide A than to peptide B. This finding stresses the relevance of peptide A over peptide B for immunodominance, and it also correlates with inhibition of parasitemia. As published in other studies in *T. gondii*, MIC-1 is also a candidate antigen for serodiagnosis, which should be investigated in *Babesia bigemina* for MIC-1 [29].

## 5. Conclusions

In this work it was shown that *Babesia bigemina* contains a *mic-1* gene. The predicted protein contains a sialic acid-binding MAR domain as well as conserved, immunodominant B-cell epitopes, which induce neutralizing antibodies. These findings are consistent with proposed ideal characteristics for the development of bovine babesiosis vaccines and/or serodiagnostic methods for this parasite species [30]. 

## Figures and Tables

**Table 1:** Presence of anti-Micronemal Protein 1 antibodies in cattle naturally infected with *Babesia bigemina*. Serum samples from *B. bigemina*-infected cattle analyzed by an indirect enzyme-linked immunosorbent assay (ELISA).

State	Farm/Ranch	Peptide A			Peptide B		
		Positive	Negative	Total	Positive	Negative	Total
Aguascalientes	A	7	2	38	9	0	38
	B	24	1		25	0	
	C	4	0		4	0	
Queretaro	A	4	0	10	3	1	10
	B	6	0		5	1	
Sinaloa	A	7	0	26	4	3	26
	B	1	0		1	0	
	C	3	0		3	0	
	D	11	0		9	2	
	E	4	0		3	1	
Veracruz	A	2	0	42	2	0	42
	B	3	0		1	2	
	C	2	0		1	1	
	D	3	0		2	1	
	E	4	0		3	1	
	F	6	0		5	1	
	G	3	0		1	2	
	H	16	0		13	3	
	I	3	0		2	1	
Total positive/negative		113	3		96	20	
